# Bird-Window Collisions at a West-Coast Urban Park Museum: Analyses of Bird Biology and Window Attributes from Golden Gate Park, San Francisco

**DOI:** 10.1371/journal.pone.0144600

**Published:** 2016-01-05

**Authors:** Logan Q. Kahle, Maureen E. Flannery, John P. Dumbacher

**Affiliations:** Department of Ornithology and Mammalogy, Institute of Biodiversity Science and Sustainability, The California Academy of Sciences, Golden Gate Park, 55 Music Concourse Drive, San Francisco, CA, 94118, United States of America; University of Southern California, UNITED STATES

## Abstract

Bird-window collisions are a major and poorly-understood generator of bird mortality. In North America, studies of this topic tend to be focused east of the Mississippi River, resulting in a paucity of data from the Western flyways. Additionally, few available data can critically evaluate factors such as time of day, sex and age bias, and effect of window pane size on collisions. We collected and analyzed 5 years of window strike data from a 3-story building in a large urban park in San Francisco, California. To evaluate our window collision data in context, we collected weekly data on local bird abundance in the adjacent parkland. Our study asks two overarching questions: first–what aspects of a bird’s biology might make them more likely to fatally strike windows; and second, what characteristics of a building’s design contribute to bird-window collisions. We used a dataset of 308 fatal bird strikes to examine the relationships of strikes relative to age, sex, time of day, time of year, and a variety of other factors, including mitigation efforts. We found that actively migrating birds may not be major contributors to collisions as has been found elsewhere. We found that males and young birds were both significantly overrepresented relative to their abundance in the habitat surrounding the building. We also analyzed the effect of external window shades as mitigation, finding that an overall reduction in large panes, whether covered or in some way broken up with mullions, effectively reduced window collisions. We conclude that effective mitigation or design will be required in all seasons, but that breeding seasons and migratory seasons are most critical, especially for low-rise buildings and other sites away from urban migrant traps. Finally, strikes occur throughout the day, but mitigation may be most effective in the morning and midday.

## Introduction

Each year, between 365 million and a billion birds die from window collisions in the United States of America alone [1–3], suggesting that bird-window collisions are the second largest anthropogenic cause of bird mortality, behind outdoor domestic cats. These strikes are a major conservation issue [3–6] and many species–including vulnerable or declining species–are susceptible to collisions [1].

Due to concerns about impacts on avian populations and preventing window collisions, research has been conducted to understand why birds strike windows [1, 4, 6]. In order to understand why collisions occur, we asked two questions: first, what aspects of a bird’s biology makes them more likely to fatally strike windows; and second, what characteristics of a building’s design tend to cause bird strikes.

Multiple aspects of a bird’s biology have been implicated in fatal window strikes. For example, Hager et. al [7] found that juveniles were more susceptible to striking than adults. Klem et al. [4] found no significant difference in the age or sex of the birds or the seasonality of strikes. O’Connell [8] found that window strikes peaked during migration, suggesting that birds are highly susceptible along their migratory flyways. Nocturnal migrants are especially susceptible to striking tall communication towers [9, 10], indicating that high-rise buildings may have qualitatively different dynamics of which birds strike and when. There may be many aspects of bird biology and life history, such as size, territorial displays, and feeding and migratory behaviors that might affect their susceptibility to fatally strike windows.

Likewise, many characteristics of windows and building design have been implicated in increased bird strikes. Studies show that birds do not recognize clear or reflective windows as fatal barriers [2], and windows are most dangerous when the surrounding habitat and sky is clearly visible through or reflected in the glass [2]. Strikes occur more frequently on lower windows during the day due to the increased bird activity closer to the ground [11], but tall towers threaten migrants moving at night [9]. Environmental factors can also affect window strikes, including whether bird feeders or desirable avian habitat is located near windows [2, 7]. The orientation of windows to sunlight might affect glare and reflection at key times of day, thus affecting strike rates [12]. Furthermore, some characteristics of windows themselves may affect the likelihood of bird strikes, for example, strike fatalities may decrease with angled windows [2, 12], although this may be highly dependent upon which direction birds are flying and the reflections that are seen by them.

Understanding which birds strike and why is important for guiding management decisions to prevent window strikes at existing buildings and to minimize collisions at newly designed buildings. Costly mitigation efforts can be more appropriately targeted and be more effective if we know more about which birds strike, at what times of year, during which times of day, and against what types of windows. Furthermore, many places–such as San Francisco, Toronto, New York, and Chicago–have considered bird-safe building regulations for future projects [5, 13]. Such efforts are strengthened by data that can demonstrate the scale of the problem, can help elucidate the most problematic building structures, and can suggest alternative designs that reduce strikes.

Of the studies published to date, few included year-round or multi-year data, and even fewer have been conducted along western United States flyways [1]. Year-round data are important for examining seasonal differences, examining relative contributions of migrating birds and resident birds, and evaluating differences between young and adult birds. Here, we hypothesized that more birds would strike during active migration than during summer or winter, and that immature birds would be more likely to strike than adults. Multi-year data are also important for increasing sample sizes and for assessing variation among years. Additionally, there are data suggesting that the western flyways have fewer migrating birds [14], as well as a different species composition of resident birds, thus questioning the applicability of results from studies done elsewhere. Most published studies only document standardized surveys, usually conducted in the early morning, that assumes a majority of strikes occur during overnight migration [1]. These data do not address the issue of window strikes over a 24-hour period. Hager and Craig [15] determined that daily mortality was highest between sunrise and 1600h, thus highlighting the importance of documenting window strikes throughout the day. We hypothesized that window strikes would peak early in the day during peak bird activity periods.

Here, we report a continuous five-year study of window strikes from a large building with significant glass exterior and a living roof. The building is the California Academy of Sciences (CAS), a 3-story public natural history museum, aquarium, and planetarium on the west coast flyway. The building was recently rebuilt and opened to the public in October 2008 in Golden Gate Park, a 412-hectare park in San Francisco, California. Golden Gate Park, a small strip of park habitat in a large city, attracts a variety of migrant bird species as well as residents. The glass exterior of CAS poses a potential collision threat for birds utilizing parkland habitat surrounding the building and the habitat provided by the living roof. Window strikes were first noticed shortly after museum staff moved into the building in the Spring of 2008. We have since accumulated data and specimens from over 355 total strikes (308 documented fatal strikes), involving more than 30 species, averaging about 60 fatal strikes per year. This number is relatively high for a single building of this size given data from other parts of the country [1]. Loss et al. [1] additionally noted the lack of studies from the western flyway, and used some of our preliminary data for their analyses. Our multi-year year-round study will provide a useful comparison between the strikes in Eastern and Western North America

As a museum, we were able to collect and prepare voucher specimens of all bird carcasses that were recovered after building strikes. Thus, we could document the age, sex, and species of most birds that died. We also documented where and when they struck the building. This allowed us to evaluate a number of hypotheses about the timing of strikes including seasonality and time of day and whether there were differences in species, sex, age, or migrant status of birds that struck windows. We hypothesized that males would be over-represented due to more aggressive and territorial tendencies and increased movement. Juveniles were predicted to be more susceptible than adults due to lack of experience with the area and the windows. Similarly, we predicted migratory birds would be more susceptible than residents due to unfamiliarity. While we were uncertain if any particular side of the building would experience proportionally more strikes than the other sides, we hypothesized that strikes would occur in proportion to window area. In order to provide a comparison to expected values for some of these variables, we completed a full year of weekly area search surveys of birds on each side of the building and the living roof. In addition, the building had different window types that allowed us to address various impacts of window construction, including pane size and total window area. Finally, midway through the study, we utilized external window shades on some windows to reduce window strikes, allowing us to assess the effectiveness of this measure.

## Methods

### Ethics statement

No birds were intentionally harmed or disturbed during the course of this study. All surveys were done from established trails or recreational spaces on public land in Golden Gate Park following standard guidelines for the use of wild birds in research [16]. The Institutional Animal Care and Use Committee at CAS reviewed and approved the salvaging of window collision casualties under protocol number 2012–03. Dead birds were labeled and accessioned into the CAS Ornithology collection as soon as possible after they were found. Any injured or stunned birds found under windows were transferred to the Steinhart Aquarium veterinarian to evaluate, treat, and release or euthanize. If injured or stunned birds died in the vet’s care, he returned the carcasses and they were accessioned into the collections. Carcasses were salvaged under California Department of Fish and Wildlife Scientific Collecting Permit (SC-7293) and federal U.S. Fish and Wildlife Service Scientific Collecting permit (MB-680765-1).

### Study location

We studied window strikes at CAS, a public museum, aquarium, and planetarium located in Golden Gate Park, San Francisco, California (latitude and longitude 37.77 x -122.466). This Double Platinum LEED-certified building is rectangular in shape with a roof area of approximately 1.5 hectares including overhang, and is three stories tall above the ground level. The building is topped with a living roof and planted with native Californian plants. The building was under construction from 2004 through 2008, and officially opened to the public in October 2008.

**Fig 1:**
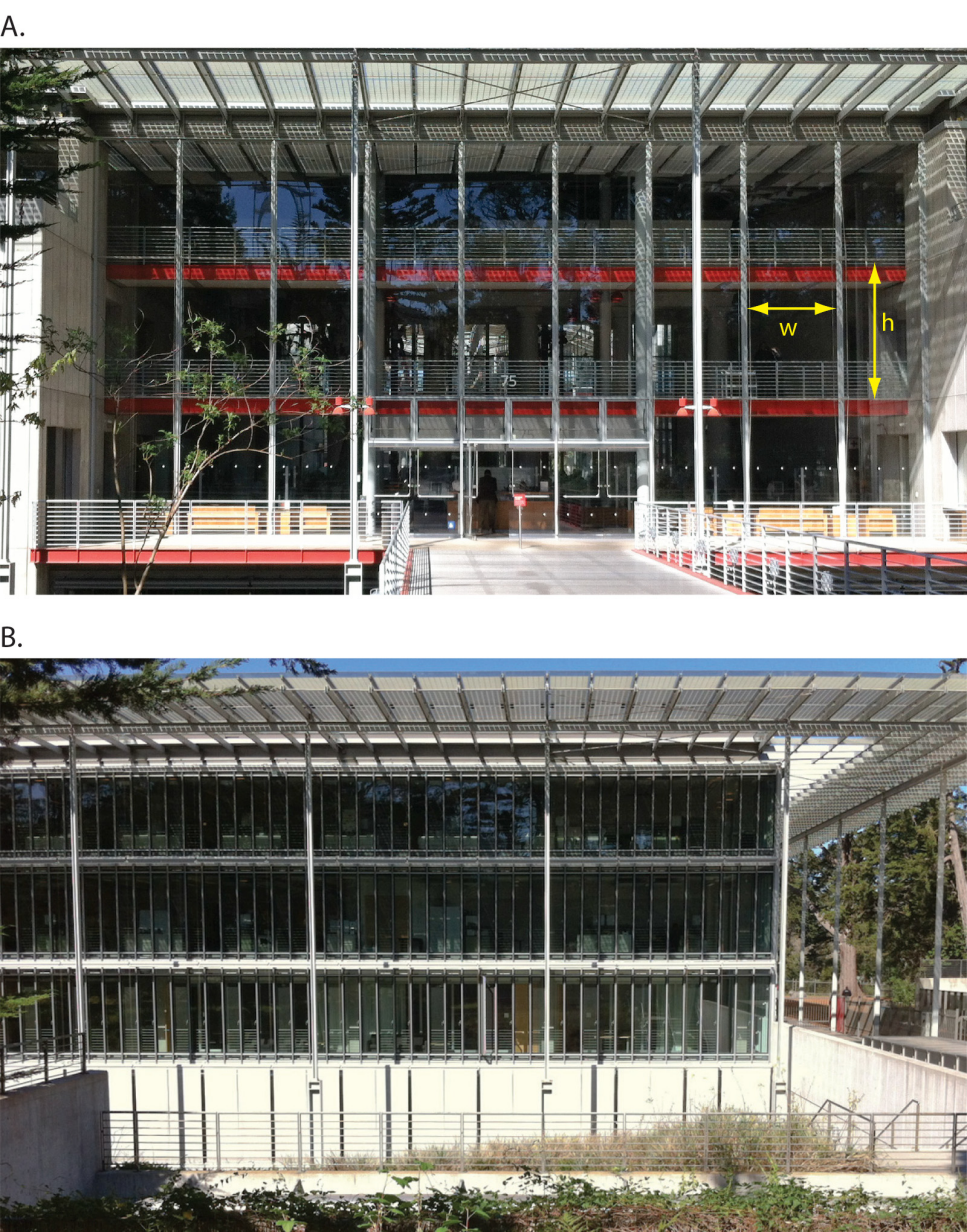
Photos of the different window pane types found at CAS. (A) shows the large panes at the south side business entrance. (B) shows the bank of small pane windows on the south side of the building with panes less than 0.5 m wide.

As part of the initial design, the building has extensive exterior windows on all four sides to allow natural light to enter, thus reducing the need for electrical lighting and heat. Window dimensions were measured by hand and the numbers of windows and their sizes were counted and confirmed using the designers’ building plan. Windows were divided into two main types: small panes (0.5 m or less in width) and large panes (1 m or larger in width; Fig 1). The east and west sides of the building and the north and south entrances are composed of many large panes, each approximately 3.4 m high by 2.3 m wide, or about 7.8 m^2^. The large pane windows are separated from each other by 15 cm wide metal mullions. Together, these large panes present a wall of windows with a combined surface area of about 205 m^2^ on the north and south, and 368 m^2^ on the east and west (Table 1). The remaining south side of the building, which houses the Administrative offices, is made up of over 800 small window panes that are 0.48 m wide and separated by metal mullions, each 13 cm wide. These smaller paned windows cover a total surface area of 1237 m^2^. In general, night-time lighting is reduced building-wide to the minimum necessary security lights at each entrance and throughout interior spaces, and offices are darkened to save power. Interior lights in exhibit spaces are mostly turned off to provide darkness for aquarium exhibit plants and animals. The lighting at each side of the building and at large and small panes is qualitatively similar.

**Table 1:** Window locations, total area, number of fatal strikes per area, and an estimate of the number of strikes per unit area per day. After 812 days of the study, shades were extended over the top two-thirds of the east and west windows to mitigate bird strikes. This mitigation continued for 1016 days. East and west side mortalities were tallied for the periods pre- and post-mitigation.

Glass Window Location	Window Area [m^2^]	Fatal strikes	strikes/m^2^/day
North Public Entrance	202.33	81	2.19E-04
South Staff Entrance	205.42	38	1.01E-04
East Garden (totals)	367.85	74	
Pre-mitigation		70	2.34E-04
Post-mitigation		4	1.07E-05
West Garden (totals)	367.85	77	
Pre-mitigation		63	2.11E-04
Post-mitigation		14	3.75E-05
South Small Windows	1237.17	24	1.06E-05
Unknown Location		14	
**TOTAL**	**2380.62**	**308**	**7.08E-05**

### Strike data

We began collecting window strike data on 10 February 2008. Data were collected opportunistically until daily surveys were instituted on 03 March 2009 and continued until the end of 2013. Daily surveys were conducted in the morning before the building opened to the public when staff members were present, generally Monday through Friday, but also included some weekend days. Our standard carcass survey consisted of a single staff member searching for dead or injured birds under all large pane windows and under small paned windows on the south side of the building.

Additionally, many carcasses were found by other museum staff outside of the morning carcass surveys. To capture data about these birds, we devised a simple protocol, and all staff were informed about how to respond if they encountered a dead bird. A small freezer was designated for the study and placed where any staff member could access it. Bags and forms were provided for collecting the carcasses and recording collection data including date, time, the location where the bird was found, the collector’s name, and the tentative species identification, if known. Birds and completed forms were placed in the freezer. All birds collected were prepared as museum specimens with complete data on weight, age, sex, and are permanently housed at CAS. Strike data are available as online supporting information, S1 Data: Window Strike Data, in spreadsheet format.

### Area search surveys

To estimate relative abundance of bird species using the adjacent park, we surveyed birds using standard area search protocol [17 page 35]. We surveyed four different habitat patches, each adjacent to one side of the building. Each study area around the building was approximately 1.5 hectares to match the size of the living roof with roof overhang. Surveys were conducted primarily on Tuesdays and Thursdays within 2.5 hours of sunrise. Each survey lasted 30-minutes and covered the prescribed area as thoroughly as possible. Each area was surveyed once per week throughout calendar year 2013. We conducted a minimum of 14 and a maximum of 20 surveys in a month. Low counts were caused by cancelled surveys due to inclement weather (i.e. heavy rain). If surveys were scheduled on a day with poor weather, they were postponed and completed as soon as possible that week. If poor weather persisted into the next week, the survey was canceled for the week. To adjust for the differences in the numbers of surveys completed, we used the average numbers of birds per survey per month for analyses.

Every bird encountered within the area was identified to species, sex and age when possible, and recorded as a visual, song, or call encounter. Birds that were observed immediately outside the area or flying over were recorded, but not used in analyses. All area search survey data were entered into eBird (eBird.org), a public bird sighting database. Data were then downloaded from eBird in tabular form for analysis. Data included fields on species, age, sex, date, and location, all of which could be tallied and searched. We analyzed a full year of survey data collected from January 1, 2013 to December 31, 2013. Area search data are available as online supporting information, S2 Data: Area Search Data, in spreadsheet format.

### Hypothesis testing

We performed a variety of exploratory statistical analyses to test for correlates of a bird’s biology that might relate to strikes, including which species were most prone to striking, when birds were most likely to strike (time of year as well as time of day), and whether a bird’s sex or age affected striking.

To test hypotheses regarding which species were over- or underrepresented in fatal window strike data, we used data from the area surveys for information on the relative abundance of each species in the adjacent park. Under the null model, birds should be striking in proportion to their frequency in the environment [18]. We used the cumulative binomial distribution to assess the significance of deviations from the expected frequencies, i.e. whether particular species were significantly over- or underrepresented in the fatal strike data.

We hypothesized that migratory bird species might strike more frequently than non-migratory species due to resident birds’ familiarity with the area as well as resident birds more sedentary habits. We designated a species as “migratory” if individuals of the species are not year-round residents of Golden Gate Park. Thus, this considered only whether bird species were migratory or not, and not whether these individual birds were actively migrating through the park. To test whether or not migratory species were over or underrepresented, we ranked each species by how over- or underrepresented they were in the strike data (for ranked order and for designation of migratory or non-migratory status, see S1 Table: Table of all fatally striking bird species.) We then used the Mann-Whitney U test for ranked unpaired observations [19] to test for an association of migratory status and overrepresentation in the strike data.

We tested whether sex or age affected the probability of striking windows. Only bird carcasses from fatal strikes could be reliably aged and sexed. Consequently, only fatal strikes were used for these analyses. During specimen preparation, birds were sexed by examining and measuring gonads, as well as by examining plumage characteristics [20, 21]. Birds were aged by examining skull ossification, bill serration length (hummingbirds), gape characteristics, plumage, molt limits, and other external characteristics [20, 21]. We scored each carcass for its age class, using two age classes, Hatching-year (HY) and After-hatching-year (AHY) birds, corresponding to immature and adult birds respectively. As convention, birds become AHY as of January 1 each year. To test the hypothesis that males were more likely to strike than females, we assumed that the ratio of males to females was 50:50, and used the binomial distribution to test for deviations from expected values. To test the hypothesis that young birds were more likely to strike windows than adults, we used unpublished data from Point Blue Conservation Science (formerly Point Reyes Bird Observatory) to assess the expected ratio of HY and AHY birds in the habitat, and the binomial distribution to test for deviations from expected values.

We additionally performed a variety of exploratory statistical analyses to test for correlates of window construction and placement. To examine whether different window pane types had different effects on bird strikes, we converted the number of strikes to units of strikes per m^2^ of glass per day for the duration of the project [strikes/m^2^/day] to provide a simple comparison. To test whether there was a particular side of the building that birds were more likely to strike, we used the Chi-squared goodness of fit tests. For analyses that account for window area and orientation, we calculated the expected number of strikes for each side by multiplying the total number of birds that struck the entire building by the proportion of window area on that particular side of the building. For analyses based upon bird abundance and activity on each side of the building, we calculated the expected values by multiplying the total number of fatal strikes by the ratio of total birds observed in the adjacent area to the total number of birds in all areas.

### Mitigation efforts

**Fig 2:**
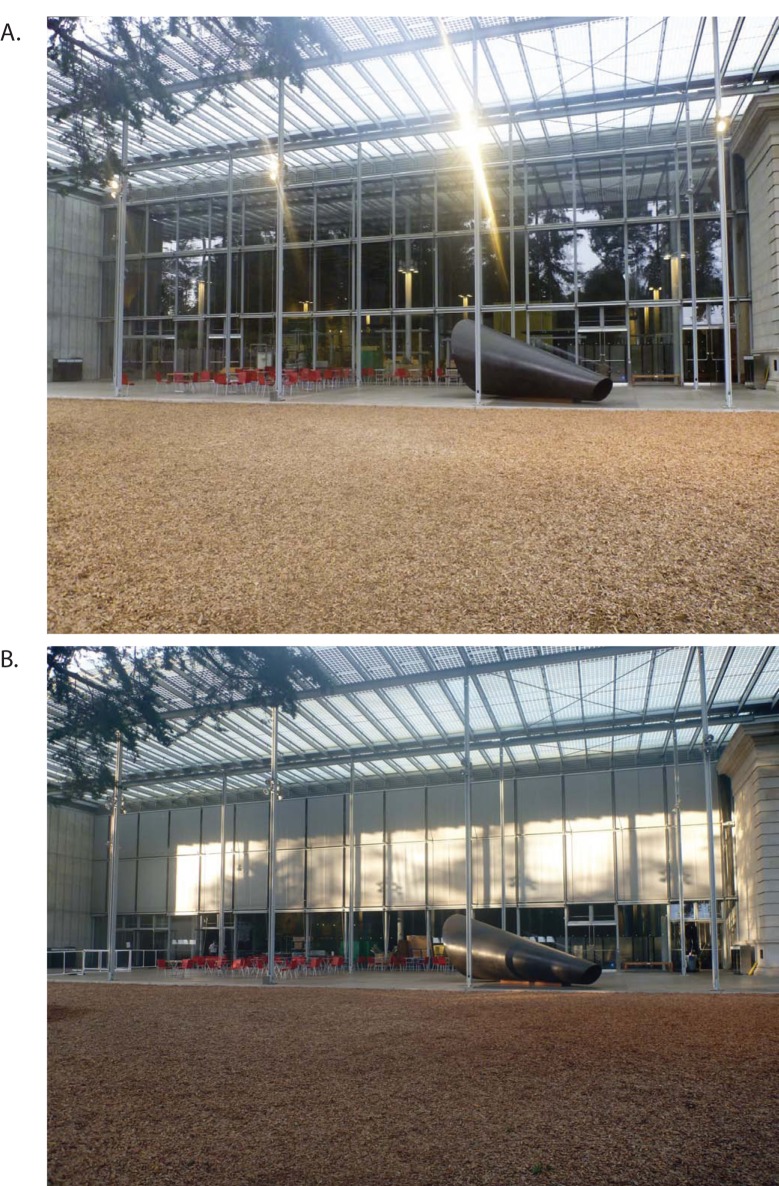
Photos of the east side windows without exterior shades (A) and with exterior shades (B). Note that the shades cover only the top 2/3rds of window area, and completely block the windows. Shades were originally designed to shade and control interior lighting.

To reduce bird strikes on the windows, we used retractable shades on the outside of the east and west large pane windows (Fig 2). These were vertical shades extending over the windows on levels 2 and 3 and effectively blocked all of the glass more than 3.5 m above the ground, which was also 2/3rds of the total window area. Shades were programmed to extend for 24 hours per day, wind speed permitting, from 22 March 2011 onward. On windy days, which were rare, the shades would automatically retract and stay retracted until wind speeds allowed for the shades to be re-extended. Thus, strikes on the east and west sides after 22 March 2011 correspond to a 2/3rds reduction in glass area.

### Carcass persistence study

The presence of scavengers may affect carcass detection and overall estimates of bird-window collisions [7, 12, 22–24]. To test how effectively we detected and recovered window strikes around the building, we set out a motion-triggered digital camera trap and a bird carcass as bait to evaluate whether window casualties were being removed or not reported. We set the camera and carcass five times on each of six windowed sections of the building, the east and west sides, the north and south entrances, and the Administrative office windows. The camera was set for a total of 30 nights over the course of a 70-week period. The bait carcass was an uncatalogued specimen, either a passerine (n = 27) or a hummingbird (n = 3), placed on the bare ground below a window and within 1m of the window. The specimen was set between 1700 h and 2000 h, and if not removed, retrieved the next morning, usually between 0800 h and 1000 h. The average duration that the camera trap and carcass were deployed was 14.75 hours.

We used a Bushnell 8MP Trophy Cam HD Hybrid Trail Camera with Night Vision programmed to include the date, time, and temperature on each image. Once activated by motion, the camera took three pictures at five second intervals. A manufacturer’s setting on the camera rendered it inoperable for one minute after taking the third picture. The camera was mounted on a stanchion within one foot of the ground and 15–20 feet from the carcass, depending on the space available. The camera and stanchion were removed after the morning survey and all images were downloaded. During morning surveys, we recorded a carcass as being removed if we did not locate body parts containing flesh, bones, or more than 10 disarticulated feathers and photos included (1) images of the scavenger with the bird in its mouth, (2) an initial image of the scavenger and the carcass in the same frame followed by an image of the scavenger only with the carcass missing, or (3) an image of the scavenger only with the specimen missing. We recorded a carcass as a reported window collision if (1) any CAS staff member, other than the staff member who set up the camera and carcass, collected the specimen or (2) if any staff members reported the carcass directly to Ornithology and Mammalogy staff or to the CAS Receptionist, or (3) it remained on the ground when we performed our standard morning window surveys.

## Results

### Area survey data

We recorded 6280 bird-observations during 202 area surveys conducted during 2013, documenting 72 species inhabiting or using the areas immediately adjacent to the CAS building. Data from these surveys provided information of which bird species were present in the area and might be exposed to the building and its glazed windows, and were used to calculate expectations for various fatal window strike probabilities.

### Window strike overview by species

Throughout the study (10 Feb 2008 to 31 December 2013), 355 birds struck the windows and were stunned enough to be found and counted. Of these, 308 resulted in mortalities (87%), while the remaining 47 were released with a good prognosis of survival. 40 species, four of which never struck fatally, were documented among these strikes (see Table 2 and supplemental materials). Using the binomial expectation to identify species that fatally struck more often than expected, 14 species were significantly more abundant in window strikes than in the adjacent bird populations (Table 2). Hummingbirds struck most frequently with Anna’s Hummingbird (*Calypte anna*) accounting for over 42% of all strikes (*n* = 131, P<0.001). *Selasphorus* hummingbirds, both Allen’s Hummingbird (*Selasphorus sasin*) and Rufous Hummingbird (*S*. *rufus*), were the second most frequently represented species (*n* = 42, one *Selasphorus* specimen could not be identified to species, and appears in the tally in Table 2 as “unknown species”, P<0.001). We found that migratory species were over represented in comparison to year-long residents (Mann-Whitney U test for large samples and multiple ties [19], t_s_ = 3.629, P<0.01).

**Table 2:** A list of bird species fatally striking the windows at CAS. Probability of *n* strikes is the cumulative binomial probability of *n* strikes, which indicates if birds are over-represented in window strike data (P<0.05) or under-represented (P>0.95). Some birds that did not fatally strike were included if they were very common in area surveys, and they were significantly under-represented in the strike data (P>0.95). Four species of birds struck the windows, but were never fatally injured (*Buteo jamaicensis*, *Accipiter cooperii*, *Charadrius vociferus*, and *Troglodytes pacificus*.) See supplemental materials for more information.

Species	Number of fatal strikes (*n*)	Probability of *n* strikes
*Calypte costae*	1	<0.001
*Passerculus sandwichensis*	2	<0.001
*Geothlypis trichas*	3	<0.001
*Selasphorus sasin*	37	<0.001
*Selasphorus rufus*	4	<0.001
*Calypte anna*	131	<0.001
*Zenaida macroura*	6	<0.001
*Setophaga petechia*	7	<0.001
*Catharus ustulatus*	1	0.001
*Melospiza lincolnii*	6	0.002
*Cardellina pusilla*	3	0.002
*Catharus guttatus*	8	0.020
*Empidonax difficilis*	1	0.025
*Vireo gilvus*	1	0.025
*Setophaga coronata*	7	0.083
*Sayornis nigricans*	3	0.086
*Columba livia*	1	0.166
*Oreothlypis celata*	2	0.169
*Molothrus ater*	1	0.567
*Passerella iliaca*	6	0.590
*Spinus psaltria*	1	0.632
*Junco hyemalis*	22	0.680
*Certhia americana*	1	0.721
*Setophaga townsendi*	3	0.726
*Melozone crissalis*	1	0.939
*Haemorhous mexicanus*	5	0.949
*Sturnus vulgaris*	1	0.960
*Haemorhous purpureus*	0	0.960
*Bombycilla cedrorum*	0	0.973
*Zonotrichia atricapilla*	3	0.996
*Regulus calendula*	0	0.999
*Spinus pinus*	0	0.999
*Poecile rufescens*	1	>0.999
*Sitta pygmaea*	1	>0.999
*Zonotrichia leucophrys*	1	>0.999
*Turdus migratorius*	3	>0.999
*Agelaius phoeniceus*	1	>0.999
*Euphagus cyanocephalus*	25	>0.999
*Melospiza melodia*	5	>0.999
*Aphelocoma californica*	0	>0.999
*Psaltriparus minimus*	1	>0.999
Unknown species	2	
TOTAL	308	

In addition, 15 species were determined to be significantly underrepresented in window strikes because they were detected in larger relative proportions in the habitat surveys than in window strikes. These include five species that were not observed striking the windows at all (Table 2). Two species were underrepresented despite significant numbers of strikes, because they were common in the habitat. These included Brewer’s Blackbirds (*Euphagus cyanocephalus*) with 25 fatal strikes and Dark-eyed Juncos (*Junco hyemalis*) with 22 fatal strikes.

### Sex of birds striking

For comparisons of sex and age classes in window strikes, we pooled all fatal strike data from all years, for a total of 308 observed mortalities. Of the 277 birds that were sexed (31 were left undetermined), 93 (34%) were female and 184 (66%) were male (see Table 3). Assuming there was an equal number of males and females in the perimeter, males were significantly overrepresented (binomial probability, P = 2.44 x 10^−8^). Also, similar binomial tests were conducted independently for each month to test whether the sex bias differed throughout the year (see Table 3.) Even if all birds of unknown sex were scored as females, there is no month of the year that we observed more females than males striking windows, and August through October had the highest ratio of male to female strikes with a ratio of 2.5 males to each female during this period.

**Table 3:** Number of fatal window kills by month and sex

Month	Females	Males	Unk	Total
January	4	8		12
February	1	*7	1	9
March	5	6		11
April	7	15	3	25
May	5	*14	5	24
June	11	14	1	26
July	15	21	4	40
August	8	*21	3	32
September	8	*21	4	33
October	14	**32	9	55
November	10	19	1	30
December	5	6		11
**Total**	**93**	**184	**31**	**308**

We used * to indicate where observed numbers of males were significantly higher than expected based upon the binomial distribution. We assumed a 50:50 ratio of males to females in the areas adjacent to the building (** P<0.01, *P<0.05).

### Age of birds striking

For comparisons of age classes in window strikes, 64 of 308 birds were classified as unknown age class (mostly late year birds or hummingbirds.) 244 fatal strikes were assigned to age class, with 148 HY birds and 96 AHY birds recorded (Table 4). To evaluate whether HY birds struck windows more often than randomly expected, we used monthly banding data from Point Blue’s Palomarin Field station in nearby Marin County, CA, during this same period (2008 through 2013) to estimate the ratio of HY to AHY birds in the environment, and we used the binomial probability test to test for significant deviations from expectation. Although fewer HY than AHY birds struck in April, HY birds were still significantly overrepresented since they should be so rare in the habitat in April. From May through October, more HY birds struck than AHY birds, and numbers of HY birds were greater than expected in April through July (binomial probability test, P<0.01, Table 4). We recorded over 10 times more HY than AHY birds in August and September, and although this represented more HY than expected, the deviation was not statistically significant. The ratio of HY to AHY birds dropped drastically in October, November, and December (Table 4), and in fact AHY birds were statistically overrepresented, however this may be due to the large numbers of birds that could not be reliably aged at this time of year, many of which were likely HY.

**Table 4:** Number of fatal window kills by month and age. We used ** to indicate where observed numbers were significantly higher than expected based upon banding data from nearby Palomarin field station (binomial probability < 0.01).

Month	AHY	HY	Unk	Totals
January	12			12
February	9			9
March	11			11
April	16	**5	4	25
May	8	**14	2	24
June	1	**21	4	26
July	4	**34	2	40
August	2	23	7	32
September	2	22	9	33
October	**16	19	20	55
November	**10	9	11	30
December	**5	1	5	11
**Age Totals**	**96**	**148**	**64**	**308**

Because hummingbirds represented over half of our window strikes, we excluded hummingbirds from a copy of the data and re-ran many of our analyses. The ratio was 58 HY to 30 AHY passerines with 46 individuals of unknown age. AHY birds were still significantly overrepresented (P < 2.0 x 10^−6^) overall. The sex ratio in passerines was 70 males to 42 females with 22 unknowns. Males were still significantly overrepresented (P<0.006). The overall strikes of passerines followed a similar yearlong trajectory as the dataset that included hummingbirds. The only discernible differences were a reduced peak in mid-Summer and a more obvious peak in late Fall.

### Time of day

**Fig 3:**
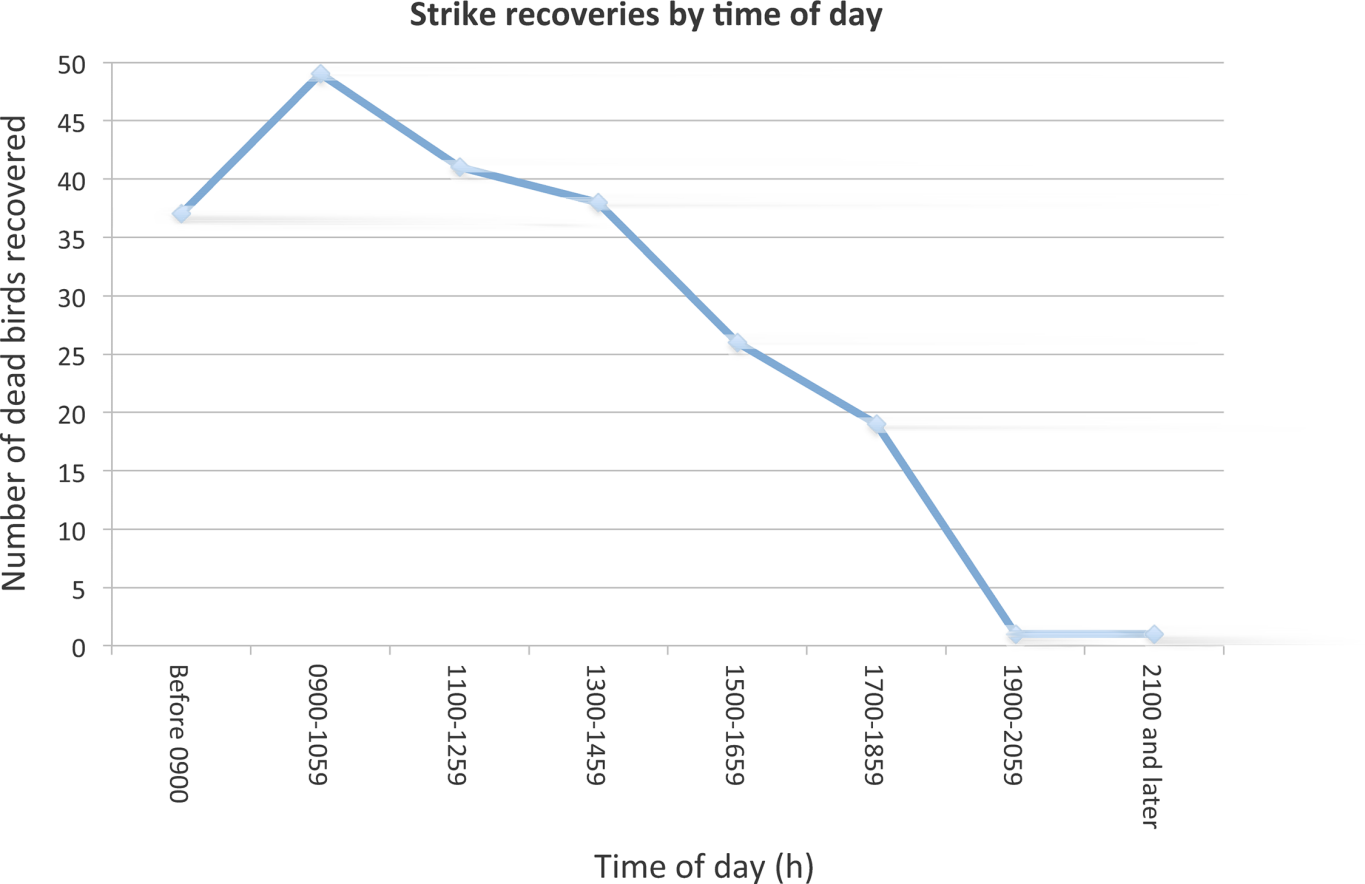
Strike recoveries by time of day. The standard survey took place prior to 0900 h and would recover any carcasses from strikes overnight. Any birds reported after 0900 h would be from incidental recoveries from other museum staff outside of our standard morning surveys.

We began recording time of day of each strike systematically in March 2009, resulting in 212 carcasses with reliable data on the time that they were found. Carcasses were found during all daylight hours (see Fig 3) with the greatest number of carcasses between 0900 h and 1100 h (n = 49), but strikes occurring at other times: before 0900 h (n = 37), and from 1100 h to 1300 h (n = 41). Strike recoveries before 0900 h were mostly collected during our standardized surveys, although these accounted for only 17% of total strikes. Another study found that most strikes occurred in early and late morning, and were as much as four times greater than at other times of the day [4]. Similarly, Hager and Craig [15] found that the majority of birds died between sunrise and 1600 h with a peak in the midday. Our study had similar results overall, with higher strike rates throughout the day but a steady decline of strikes after the morning hours.

### Time of year

**Fig 4:**
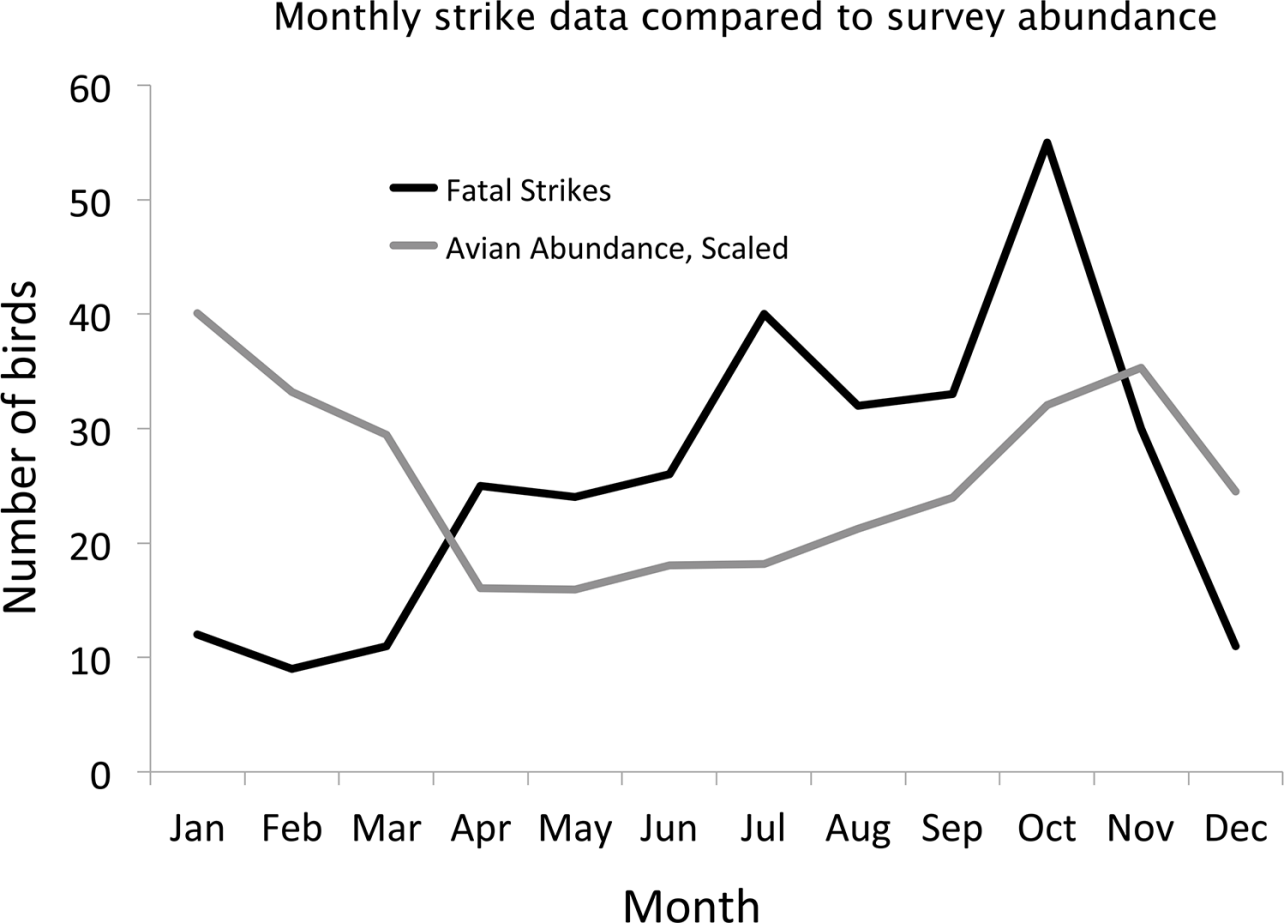
Monthly strike data compared to survey abundance. Although avian abundance is highest in November through March, fatal strikes are relatively lower during this period.

We summarize bird mortality by month (see Tables 3 and 4), and plotted those data with avian abundance from the area search survey data (Fig 4). Avian abundance was derived from the average number of birds detected per survey for each month, scaled so that totals across all months equaled the total number of fatal strikes. Thus scaled abundance could alternatively be viewed as an “expected number of strikes per month” based upon abundance, and it could be easily seen whether fatal strikes simply track the abundance of birds detected in the survey data.

Avian abundance varied throughout the year. The average number of birds detected per area survey ranged from a low of 20 birds/survey in July to a high of over 49 birds/survey in December. The bird numbers detected in the surveys remained relatively constant from October to February, but dropped steadily into April and May.

During the breeding season (April—October), fatal strikes exceeded expectations based upon avian abundance, although both generally increased as the year progressed and birds produced more young. Between November and March, fatal strikes were fewer than expected (Fig 4), despite the increase in avian abundance with the influx of winter residents. There were three distinct peaks in fatal strike numbers corresponding to April (25), July (40), and October (55).

### Total window area and type of window

The building has two window types that we classified as large pane and small pane windows. These two window types killed birds at very different rates. Overall, the small pane glass had a lower strike rate of 1.06 x 10^−5^ fatal strikes/m^2^/day. Large pane glass had an average strike rate 1.79 x 10^−4^ fatal strikes/m^2^/day–almost 17 times more fatal strikes per unit glass than the small paned glass. To control for other factors (direction, amount of light, bird species in the habitat, etc.), we also compared large and small paned glass on only the south side of the building, because the south side had both types of windows. South side large paned glass had nearly 10 times more fatal strikes (1.01 x 10^−4^ strikes/m^2^/day) than the south side small paned glass (1.06 x 10^−5^ fatal strikes/m^2^/day). Overall, CAS has approximately equal total area of the two window types with the total area of large-paned glass equaling 1143 m^2^ and the total area of small paned glass at 1237 m^2^. Nearly all (91.11%) of fatal window collisions occurred at large paned windows and only 8.89% occurred at the small paned windows (see Table 1.)

### Orientation of windows

To compare the effect of window orientation (north, south, east, west), we used only large paned window strikes during the pre-mitigation period (before shades were deployed on the east and west sides to prevent strikes). Bird-window collisions were not evenly distributed around the building by window area (chi-squared test, X^2^_df = 3_ = 12.9, P<0.005). The most significant deviation from the expected number of strikes was the paucity of strikes on the south side staff entrance. The east side had the highest strike rate, at 2.34 x 10^−4^ strikes/m^2^/day, while the north and west sides were slightly higher than the expected values (see Table 1 for strike rates).

Each side of the building differed qualitatively in habitat type, disturbance and human activity, and therefore the amount of bird activity. We derived expectations based upon the numbers of birds from area survey data on each side of the museum and found that birds did not strike windows in proportion to their abundance in the adjacent habitat (chi-squared test, X^2^_df = 3_ = 55.2, P<0.001). Fewer birds struck the north and south large windows than expected, and more birds struck the east and west sides than expected.

### Effects of mitigation

**Fig 5:**
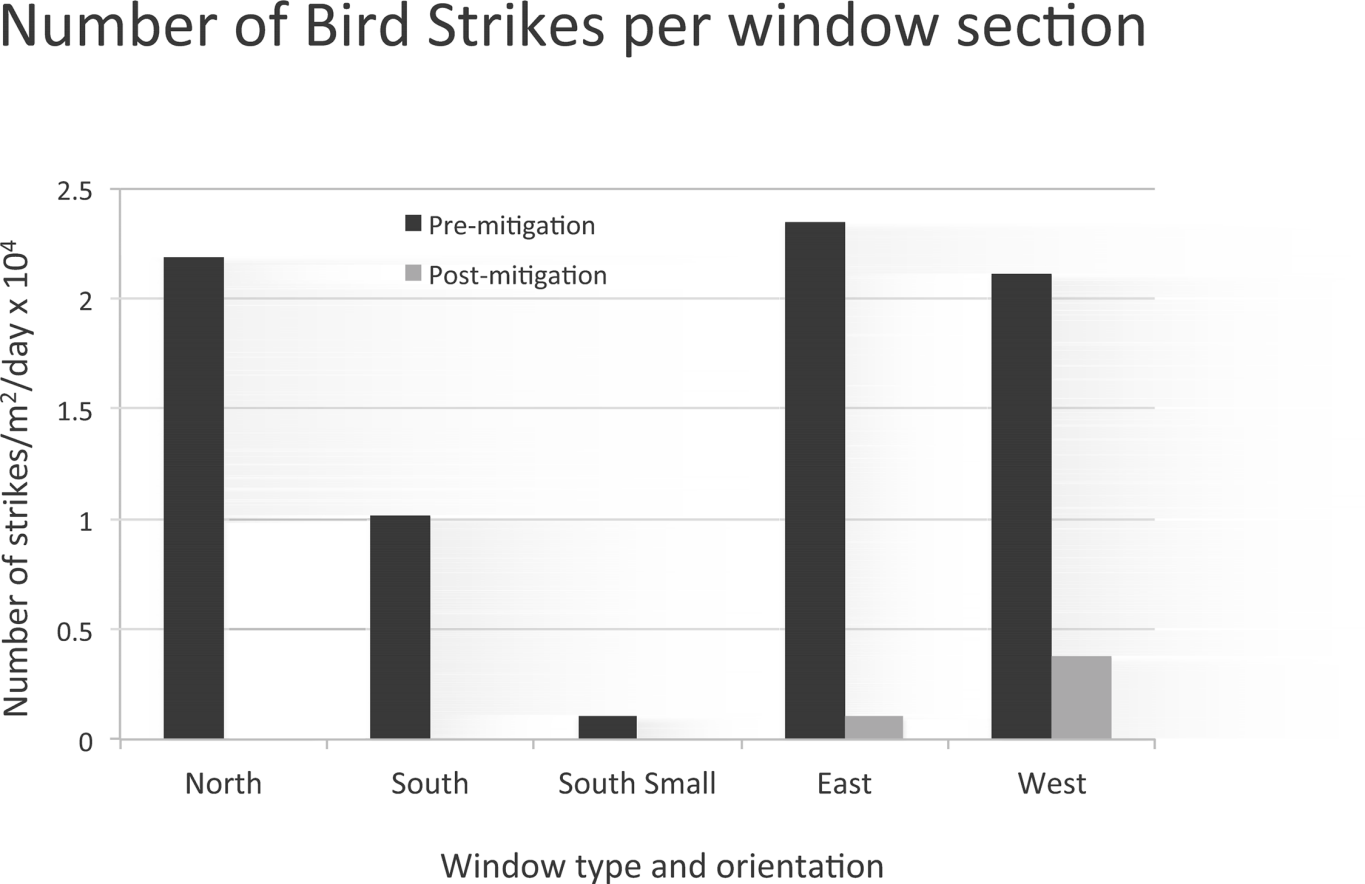
Number of bird strikes per window section, in units of strikes/m^2^/day x 10^4^. Effects of mitigation (shades deployed on the upper 2/3rds of the window area) are also shown for the east and west sides.

After shades were deployed to cover the top 2/3rds of the windows, bird strikes dropped significantly on both the east and west sides of the building, and there was a difference in response between the east and west sides (see Fig 5). The east side encountered a drastic reduction in strikes from 2.34 x 10^−4^ to 1.01 x 10^−5^ strikes/m^2^/day. Thus pre-mitigation strike rates on the east side were almost 22 times higher than post-mitigation. Mitigation reduced strikes on the west side, but only by a factor of 5.6, from 2.11 x 10^−4^ to 3.75 x 10^−5^ strikes/m^2^/day. Both east and west sides had the same amount of glass exposed before and after mitigation, thus suggesting that differences may be due to orientation, adjacent habitat, or other factors. Although the total glass area was only reduced to 2/3rd of the original area, the strikes were reduced by a much greater factor, suggesting a non-linear response to the reduction in glass area.

### Carcass persistence

We deployed the camera trap and bait carcass for a total of 441 hours and 40 minutes over 30 nights between March 25, 2013 and July 22, 2014. We recorded six removal events, four along the west side of the building, one on the east side, and one at the front entrance. Striped skunks (*Mephitis mephitis*) were the primary scavenger species, taking four of the six carcasses. Less than 10 disarticulated feathers, too few to identify a window collision, were found after only one of the carcasses was scavenged by a skunk and before custodial staff had cleaned the area. Humans (*Homo sapiens*) removed the other two carcasses, one carcass was disposed of by early morning custodial staff and one was removed by a member of the public in the middle of the night. The camera trap photographed two other species, one raccoon (*Procyon lotor*) and one domestic cat (*Felis catus*), that both visited the bait but did not remove it. We received 12 reports of carcasses from museum staff members other than the person who set and retrieved the camera. Overall the carcass recovery rate was 80% with a removal rate of 20%. 50% of the available carcasses were recovered by museum staff not involved with the study, and the others were retrieved by Ornithology staff in the morning, at the time of our standard morning surveys.

## Discussion

Window collision studies have varied immensely with respect to locality and flyways, proximity to habitat, time of year, and methods of study; however most studies, if not all, document significant numbers of window strikes [1, 7, 8, 24–27]. Our study differs from many other window strike studies in that it is one of only a few empirical studies along the western US flyway [1], the study is continuous throughout the year and for multiple years, it examines a building surrounded by woodland and park, and it uses extensive comparative data about the local bird populations. By combining data from multiple years, our sample size of fatal strikes (n = 308) was large enough to critically examine several hypotheses, including: 1) how annual cycles of territoriality, breeding, and migration might affect strike rates, 2) how the age and sex of birds affect their susceptibility to strike, and 3) how different building characteristics contribute to bird strikes.

### Differences among bird species in strike rate

As early as 1931, ornithologists realized that certain species and families were more susceptible to fatal window strikes than others [4, 28]. In our dataset, hummingbirds were highly overrepresented in the fatal strike data with 56% (n = 174) of all fatal strikes involving hummingbirds (Table 2). Researchers across the country similarly reported that hummingbirds and swifts were overrepresented in window strike data [1, 27] and could constitute over half of their total strikes [27]. Factors that may contribute to hummingbird susceptibility include their relative fragility, high flight velocities, male territoriality and aggression, and traplining (traveling long distances to undefended nectar resources) [29]. Male (n = 114) hummingbird strikes were over twice as common as females (n = 51; with n = 9 unknown sex birds; Table 3). We documented ten independent instances in which two hummingbirds struck at the same location at the same time, suggesting an aggressive interaction or chase. Six of these were male-male pairs, three were male-female pairs, and one was a male-unknown pair.

Species that occur primarily in flocks were also underrepresented in our window collision data. Several of the most underrepresented species (European Starling *Sturnus vulgaris*, Cedar Waxwing *Bombycilla cedrorum*, White-crowned Sparrow *Zonotrichia leucophrys*, Pine Siskin *Carduelis pinus*, Brewer’s Blackbird *Euphagus cyanocephalus*, and Bushtit *Psaltriparus minimus*) form flocks at least during migration and winter. We hypothesize that during the day, flocking species may be better at avoiding windows if one or more flock member detects the window and can signal to others. Because flock members can share predator vigilance activities, they may have more free time to become aware of their immediate environment and its potential threats. If flocking behavior makes birds less vulnerable to striking buildings, this may contribute to the lower numbers of strikes in winter, when many species form flocks (e.g. parids, warblers, and some sparrows). We documented more than the expected number of strikes between April and October when birds flock less, and less than the expected number of strikes between November and March (Fig 4). Conversely, none of the overrepresented species from our collision data were found in flocks near the building. Additionally, hummingbirds and locally breeding warblers were overrepresented in our study, possibly due to lack of flocking tendencies during times when they are present.

Similar to our results, Loss et al. [1] found that blackbirds were underrepresented, though that same study also found, contrary to our findings, that some parids and sparrows were overrepresented. Many parids and sparrows are seasonal flockers in winter, and parts of Loss et al.’s sparrow and parid dataset may have been collected during Summer when flocking is less common, or while migrating (when they might strike high-rise buildings or towers at night when flocking could not benefit birds in the same way as daytime ground-foraging flocks.) This may imply variation in susceptibility within families, at different times of year, and/or in other parts of the country and by building height.

Migratory species were more susceptible to striking than year-round residents. This may be because residents rarely or never leave a small area of habitat, and thus are more familiar with their territory and its hazards. Migratory species may be less familiar with the area, or may have other behavioral correlates that increase window strikes. Of the three species that were found in the strike data and not the survey data (Common Yellowthroat *Geothlypis trichas*, Costa’s Hummingbird *Calypte costae*, and Savannah Sparrow *Passerculus sandwichensis*), all were passing migrants. Other studies have concluded that migrating birds comprise the bulk of window strikes [8–10, 27], but our data from this low-rise building suggest that strikes can occur throughout the year and involve significant numbers of residents as well as migrants.

Although previous reports suggest that all birds, large and small alike, are involved in fatal strikes [2, 4], smaller species were more susceptible to fatally striking in this study. Larger birds (hawks, owls, gulls, etc.) were rarely found stunned or dead, and of the five largest birds to be documented striking CAS, including Red-tailed Hawk (*Buteo jamaicensis)*, Cooper’s Hawk (*Accipiter cooperii*), Ring-necked Pheasant (*Phasianus colchicus*), Mourning Dove (*Zenaida macroura*), and Killdeer (*Charadrius vociferus*), only Mourning Dove struck fatally. Furthermore, the smallest birds in the study (hummingbirds) had the highest mortality. Future studies may want to focus on the physics of why larger birds are less likely to strike or die in window strikes.

### Differences between sexes in strike rate

At CAS, males fatally struck windows significantly more than females (Table 3). Evaluating strikes one month at a time, males outnumbered females in every month of the year, however differences were only statistically significant in February, May, and August through October. Males may be more likely to strike because they are more aggressive, more active in defending territories, and more actively pursuing mates, resulting in greater activity levels overall.

Previous literature stated that the differences between the number of male and female strikes was not significant [4], though this is possibly due to the tendency of Klem [4] to focus on strikes during migration. Male Common Terns at Belgian wind farms struck more often than females [30], showing that the sex bias can be found in strike rates.

### Differences in the ages of birds that strike

In our data, HY birds struck windows more often than AHY birds throughout much of the year, especially shortly after fledging. This suggests that locally breeding species are susceptible to striking, and that for many buildings, window strikes may be driven by local residents rather than actively migrating birds. Hager et al. [7] also found that HY birds were highly represented in their data, but they did not test whether they were overrepresented with respect to the numbers of HY and AHY birds in the habitat.

Klem did not find differences in age classes in strike data [4], but we believe that our results are stronger for two reasons. First, earlier studies sometimes summed data over the entire year. Because all striking birds are considered AHY birds in early parts of the year and because trends shift throughout the year, an average effect is less perceptible. Second, earlier work used a baseline of three to one ratio of HY to AHY birds as a standard for testing [4], and we used more accurate monthly estimates derived from nearby banding stations (often with an even higher expected ratio than three to one).

Because HY birds are most overrepresented from April through July when HY birds are youngest, the data suggest that less experienced HY birds early in the season are more susceptible to strikes than more experienced HY birds later in the season, i.e. November or December. Although the ratio of HY to AHY strikes drops later in the year and is less statistically significant, we think that this is primarily due to the greater numbers of unknown age birds, many of which are likely HY. Later in the year, HY birds may have fully ossified skulls, and Fall HY plumages cannot be distinguished from Fall AHY plumages for many species.

### Time of day

The majority of dead birds (83%) were collected by museum staff throughout the day rather than during standardized morning surveys (17%), suggesting that bird strikes at CAS occur all day long. Our results were similar to those from Hager et al. [15], who also found strikes were concentrated during daylight hours. The strikes increase steadily through the morning, peaking around 1000 h or 1100 h, and then declining through the afternoon (Fig 3). This is different from our initial assumption that morning surveys would exploit both overnight mortality and the peak activity of birds around first light, and that strikes would be concentrated in that time period. Given our data, surveys that take place throughout a 24-hour period will provide a more accurate count of window collision casualties than those only restricted to early morning hours.

### Strikes by month and seasonality

The number of strikes with respect to the numbers of birds in surveys suggests that birds are not simply striking more when they are more common in the environment. Throughout the year, there are distinct peaks in the numbers of fatal strikes relative to the number of birds in the habitat, especially in July and October. Migration has been considered a cause of bird strikes throughout the country [4, 8, 24, 31], and our October peak coincides with large migratory movements of many species, including certain species that are overrepresented in the strike data such as Hermit Thrush (*Catharus guttatus)*, Swainson’s Thrush (*C*. *ustulatus)*, and Lincoln’s Sparrow (*Melospiza lincolnii*). The July peak, however, is not associated with migration, but may be generated by the abundance of naive fledglings and their over-susceptibility to striking windows, as July has the highest number of HY landbirds present (data from Palomarin station, Point Blue Conservation Science). During the breeding season, residents generate many strikes, possibly due to their abundance in nearby habitat. In contrast, in urban settings with minimal or no surrounding vegetation [25] and only a few urban-adapted seasonal residents, the majority of strikes may occur during migration periods, when disoriented migrant birds lose their way in the urban or suburban cityscape with taller buildings that are illuminated at night [32]. Additionally, most other studies were conducted in the eastern United States and Canada, where several factors may be qualitatively different, including the difference in scale of the migratory movements, different bird species, more urban environments, more tall buildings, etc.

### Building characteristics and window orientation

One major finding was that even large expanses of windows had significantly reduced strike rates if they were broken up with mullions every 0.5 m. Our large paned windows have almost 17 times higher strike rate per unit glass than our small paned windows. Thus, one simple solution that may significantly decrease strikes is to either design smaller windows in new buildings or apply stickers that mimic mullions to existing structures. Although we were unable to study the optimal distance of mullions for preventing strikes, our data suggests that smaller units of glass allow birds to detect and avoid the glass surface.

Distinct discrepancies were found in the number of large-pane window strikes on different sides of the building. Other studies suggest that there is no one direction or side of the building that birds tend to strike [25]. We found it difficult to explain the differences based on any single factor, but we believe that there is a complex interaction among the amount of human activity, the amount of avian activity, the proximity of avian habitat, and bird species that frequent each side, and all of these may affect strike rates. The north and south large-paned windows are located at the two busiest entrances with most bird activity further from the glass, which might explain the relative lack of strikes on those sides. The largest discrepancy between sides was due to the relative lack of strikes on the south side. That paucity could be due to extensive human traffic during the daytime, when most birds appear to strike. Both the east and west sides have more avian habitat closer to the windows (15 and 25 m respectively) than the north side (30 m) but farther than the south side (10 m) which has extensive native plantings. The west side has a restaurant with outdoor seating, and although the area is busy during the day, blackbirds and juncos feed even when people are present, and there are food scraps that may attract birds nearer to windows. The east and west sides had very different numbers of strikes post-mitigation, as the east side had a much more drastic reduction. Thus local habitat differences are likely the primary causes of differences in strike numbers on each side of the building, though one other study states that bird behavior and window related factors were the largest drivers of strikes as opposed to abundance of bird species in nearby habitat [27].

### Mitigation efforts

Mitigation efforts using exterior shades significantly reduced window strikes. The number of strikes decreases non-linearly with window area, such that reducing exposed window area to 33% of unmitigated window area actually reduced strikes to 6–10% of unmitigated strike rate. It is possible that there is an “edge effect” such that birds can detect and avoid window surfaces if they are sufficiently close to an edge (a mullion, the ground, or some other visible object.) This may explain the non-linear response as well as the reduced strike rate at our small-paned windows. Another study supported the idea that exterior shades eliminate strikes of the covered area [2]. The effectiveness of exterior shades was larger on the east than the west side, though on both sides there was a significant reduction of strikes.

Our primary findings are that reduction in pane size and exterior shades can both reduce strikes, and these tools are applicable to other buildings. For existing buildings, it is possible that even false mullions—perhaps tape, paint, or wood—could be applied to the windows to increase the visibility of windows. Future studies should seek to understand the effect of pane size and window continuity on strikes, factors that have not been thoroughly examined in other studies, that could be critical in helping building designers provide existing buildings with more cost-effective, less disruptive approaches to reducing strikes.

Our study can inform future building design and management to decrease the number of bird strikes. Understanding strike seasonality and patterns could help additionally focus efforts, especially aesthetically unpleasing mitigation efforts, to the most important times of year and implement the most successful mitigation technique. While our data only represent the strikes at our study site, our findings are relevant to other low-rise buildings that are surrounded by avian habitat. Our data show that significant numbers of strikes can occur even in low-rise buildings, and that window mortality affects all birds in virtually all seasons and all times of day.

Based on our carcass persistence study, it is possible we are only retrieving 80% of the night and early-morning strikes. We believe that our overall detection numbers are actually higher than 80% because most carcasses were collected during the mid-morning hours outside of a morning survey. Only 17% of our window collision carcasses were found during morning surveys suggesting that only a small number of strikes occur during night and early-morning hours and even fewer would be removed by predators (see Fig 3). Thus, if we estimated overall window strikes with the addition of 20% more early morning strikes, the extrapolated number of total strikes during the five-year period would be approximately 319 window kills rather than 308. Alternatively, if carcass removal continues throughout the day at the same level (and we have no evidence for or against), then we estimate actual strike numbers at approximately 370 window kills. While our data are relatively complete, there may be additional undetected strikes.

## Supporting Information

S1 Data Window Strike Data. (XLSX)

S2 Data Area Search Data. (XLSB)

S1 Table Table of all fatally striking bird species. (DOCX)

S2 Table Table of bird species that struck windows non-fatally. (DOCX)
