# Supplementary material for: Bird-Window Collisions at a West-Coast Urban Park Museum: Analyses of Bird Biology and Window Attributes from Golden Gate Park, San Francisco
Source: PLoS One. 2016 Jan 5;11(1):e0144600. doi: 10.1371/journal.pone.0144600 (PMC4701451; doi:10.1371/journal.pone.0144600)
Supplement: S1 Table — (DOCX) [file pone.0144600.s003.docx]

S1 Table: Table of all fatally striking bird species. Note that there is one unknown bird (reported by staff, but not collected) and one *Selasphorus* hummingbird that could not reliably be identified to species. For this reason, Rufous (n=4), Allen’s (n=37), and combined (Rufous + Allen’s + 1 unknown, total n=42) are each listed separately.

| Common Name | Family | Species | Number fatal strikes | # in area surveys | Proportion of species in surveys | P(Under-represented) | P(over-represented) | Significance (*=P<0.05, ** = P<0.01, + overrepresented, - under-represented.) |
| --- | --- | --- | --- | --- | --- | --- | --- | --- |
| Anna's Hummingbird | Trochilidae | *Calypte anna* | 131 | 256 | 0.040609137 | 1 | 0 | **+ |
| Costa's Hummingbird | Trochilidae | *Calypte costae* | 1 | 0 | 0 | 1 | 0 | **+ |
| Common Yellowthroat | Parulidae | *Geothlypis trichas* | 3 | 0 | 0 | 1 | 0 | **+ |
| Savannah Sparrow | Emberizidae | *Passerculus sandwichensis* | 2 | 0 | 0 | 1 | 0 | **+ |
| Rufous Hummingbird | Trochilidae | *Selasphorus rufus* | 4 | 0 | 0 | 1 | 0 | **+ |
| Rufous/Allen's Hummingbird* | Trochilidae | *Selasphorus rufus/sasin* | 42 | 13 | 0.006662437 | 1 | 0 | **+ |
| Allen's Hummingbird | Trochilidae | *Selasphorus sasin* | 37 | 29 | 0.004600254 | 1 | 0 | **+ |
| Mourning Dove | Columbidae | *Zenaida macroura* | 6 | 3 | 0.000475888 | 1 | 2.32244E-10 | **+ |
| Yellow Warbler | Parulidae | *Setophaga petechia* | 7 | 18 | 0.00285533 | 0.999996297 | 3.70283E-06 | **+ |
| Swainson's Thrush | Turdidae | *Catharus ustulatus* | 1 | 1 | 0.000158629 | 0.998855497 | 0.001144503 | **+ |
| Lincoln's Sparrow | Emberizidae | *Melospiza lincolnii* | 6 | 35 | 0.00555203 | 0.998170568 | 0.001829432 | **+ |
| Wilson's Warbler | Parulidae | *Cardellina pusilla* | 3 | 11 | 0.001744924 | 0.99778806 | 0.00221194 | **+ |
| Hermit Thrush | Turdidae | *Catharus guttatus* | 8 | 82 | 0.013007614 | 0.979603901 | 0.020396099 | *+ |
| Pacific-slope Flycatcher | Tyrannidae | *Empidonax difficilis* | 1 | 5 | 0.000793147 | 0.974812179 | 0.025187821 | *+ |
| Warbling Vireo | Vireonidae | *Vireo gilvus* | 1 | 5 | 0.000793147 | 0.974812179 | 0.025187821 | *+ |
| Great Blue Heron | Ardeidae | *Ardea herodias* | 0 | 1 | 0.000158629 | 0.952463873 | 0.047536127 | *+ |
| Great Horned Owl | Strigidae | *Bubo virginianus* | 0 | 1 | 0.000158629 | 0.952463873 | 0.047536127 | *+ |
| Red-shouldered Hawk | Accipitridae | *Buteo lineatus* | 0 | 1 | 0.000158629 | 0.952463873 | 0.047536127 | *+ |
| Evening Grosbeak | Fringillidae | *Coccothraustes vespertinus* | 0 | 1 | 0.000158629 | 0.952463873 | 0.047536127 | *+ |
| Warbler sp | Parulidae | *Parulinae sp.* | 0 | 1 | 0.000158629 | 0.952463873 | 0.047536127 | *+ |
| Blue-gray Gnatcatcher | Polioptilidae | *Polioptila caerulea* | 0 | 1 | 0.000158629 | 0.952463873 | 0.047536127 | *+ |
| Chipping Sparrow | Emberizidae | *Spizella passerina* | 0 | 1 | 0.000158629 | 0.952463873 | 0.047536127 | *+ |
| Hummingbird sp. | Trochilidae | *Trochilidae sp.* | 0 | 1 | 0.000158629 | 0.952463873 | 0.047536127 | *+ |
| Yellow-rumped Warbler | Parulidae | *Setophaga coronata* | 7 | 92 | 0.014593909 | 0.916534519 | 0.083465481 |  |
| Black Phoebe | Tyrannidae | *Sayornis nigricans* | 3 | 34 | 0.005393401 | 0.913839228 | 0.086160772 |  |
| Band-tailed Pigeon | Columbidae | *Patagioenas fasciata* | 0 | 2 | 0.000317259 | 0.907180419 | 0.092819581 |  |
| Black-headed Grosbeak | Cardinalidae | *Pheucticus melanocephalus* | 0 | 2 | 0.000317259 | 0.907180419 | 0.092819581 |  |
| Violet-green Swallow | Hirundinidae | *Tachycineta thalassina* | 0 | 2 | 0.000317259 | 0.907180419 | 0.092819581 |  |
| Killdeer | Charadriidae | *Charadrius vociferus* | 0 | 3 | 0.000475888 | 0.864043219 | 0.135956781 |  |
| Barn Swallow | Hirundinidae | *Hirundo rustica* | 0 | 3 | 0.000475888 | 0.864043219 | 0.135956781 |  |
| Acorn Woodpecker | Picidae | *Melanerpes formicivorus* | 0 | 3 | 0.000475888 | 0.864043219 | 0.135956781 |  |
| Black-throated Gray Warbler | Parulidae | *Setophaga nigrescens* | 0 | 3 | 0.000475888 | 0.864043219 | 0.135956781 |  |
| Rock Pigeon | Columbidae | *Columba livia (Domestic type)* | 1 | 15 | 0.002379442 | 0.83364391 | 0.16635609 |  |
| Orange-crowned Warbler | Parulidae | *Oreothlypis celata* | 2 | 29 | 0.004600254 | 0.830852947 | 0.169147053 |  |
| Varied Thrush | Turdidae | *Ixoreus naevius* | 0 | 4 | 0.000634518 | 0.822950866 | 0.177049134 |  |
| Western Gull | Laridae | *Larus occidentalis* | 0 | 4 | 0.000634518 | 0.822950866 | 0.177049134 |  |
| American Goldfinch | Fringillidae | *Spinus tristis* | 0 | 4 | 0.000634518 | 0.822950866 | 0.177049134 |  |
| Zonotrichia sp. | Emberizidae | *Zonotrichia sp.* | 0 | 4 | 0.000634518 | 0.822950866 | 0.177049134 |  |
| White-throated Sparrow | Emberizidae | *Zonotrichia albicollis* | 0 | 6 | 0.000951777 | 0.74651873 | 0.25348127 |  |
| Common Raven | Corvidae | *Corvus corax* | 0 | 8 | 0.001269036 | 0.677164317 | 0.322835683 |  |
| Pacific Wren | Troglodytidae | *Troglodytes pacificus* | 0 | 8 | 0.001269036 | 0.677164317 | 0.322835683 |  |
| Northern Flicker | Picidae | *Colaptes auratus* | 0 | 10 | 0.001586294 | 0.614234162 | 0.385765838 |  |
| Hairy Woodpecker | Picidae | *Picoides villosus* | 0 | 10 | 0.001586294 | 0.614234162 | 0.385765838 |  |
| Western Tanager | Cardinalidae | *Piranga ludoviciana* | 0 | 11 | 0.001744924 | 0.584990577 | 0.415009423 |  |
| Golden-crowned Kinglet | Regulidae | *Regulus satrapa* | 0 | 11 | 0.001744924 | 0.584990577 | 0.415009423 |  |
| Nuttall's Woodpecker | Picidae | *Picoides nuttallii* | 0 | 13 | 0.002062183 | 0.530601625 | 0.469398375 |  |
| Tricolored Blackbird | Icteridae | *Agelaius tricolor* | 0 | 15 | 0.002379442 | 0.481254503 | 0.518745497 |  |
| Brown-headed Cowbird | Icteridae | *Molothrus ater* | 1 | 39 | 0.006186548 | 0.433163133 | 0.566836867 |  |
| Hooded Oriole | Icteridae | *Icterus cucullatus* | 0 | 18 | 0.00285533 | 0.415679747 | 0.584320253 |  |
| Fox Sparrow | Emberizidae | *Passerella iliaca* | 6 | 152 | 0.023635787 | 0.410124042 | 0.589875958 |  |
| Red-tailed Hawk | Accipitridae | *Buteo jamaicensis* | 0 | 19 | 0.003013959 | 0.395864726 | 0.604135274 |  |
| Lesser Goldfinch | Fringillidae | *Spinus psaltria* | 1 | 44 | 0.006979695 | 0.367734113 | 0.632265887 |  |
| Downy Woodpecker | Picidae | *Picoides pubescens* | 0 | 23 | 0.003648477 | 0.325585308 | 0.674414692 |  |
| Dark-eyed Junco | Emberizidae | *Junco hyemalis* | 22 | 510 | 0.080901015 | 0.320477025 | 0.679522975 |  |
| Brown Creeper | Certhiidae | *Certhia americana* | 1 | 52 | 0.008248731 | 0.279444593 | 0.720555407 |  |
| Townsend's Warbler | Parulidae | *Setophaga townsendi* | 3 | 101 | 0.016021574 | 0.274390302 | 0.725609698 |  |
| Steller's Jay | Corvidae | *Cyanocitta stelleri* | 0 | 30 | 0.004758883 | 0.231201759 | 0.768798241 |  |
| Hutton's Vireo | Vireonidae | *Vireo huttoni* | 0 | 37 | 0.005869289 | 0.164116217 | 0.835883783 |  |
| California Quail | Odontophoridae | *Callipepla californica* | 0 | 49 | 0.007772843 | 0.091119354 | 0.908880646 |  |
| California Towhee | Emberizidae | *Melozone crissalis* | 1 | 92 | 0.014593909 | 0.060801302 | 0.939198698 |  |
| House Finch | Fringillidae | *Haemorhous mexicanus* | 5 | 213 | 0.033788071 | 0.051388461 | 0.948611539 |  |
| European Starling | Sturnidae | *Sturnus vulgaris* | 1 | 102 | 0.016180203 | 0.040435321 | 0.959564679 | *- |
| Purple Finch | Fringillidae | *Haemorhous purpureus* | 0 | 66 | 0.010469543 | 0.039514268 | 0.960485732 | *- |
| Cedar Waxwing | Bombycillidae | *Bombycilla cedrorum* | 0 | 74 | 0.011738579 | 0.026647443 | 0.973352557 | *- |
| Golden-crowned Sparrow | Emberizidae | *Zonotrichia atricapilla* | 3 | 230 | 0.036484772 | 0.003759324 | 0.996240676 | *- |
| Ruby-crowned Kinglet | Regulidae | *Regulus calendula* | 0 | 135 | 0.021097716 | 0.001435417 | 0.998564583 | **- |
| Pine Siskin | Fringillidae | *Spinus pinus* | 0 | 153 | 0.024270305 | 0.000529863 | 0.999470137 | **- |
| Chestnut-backed Chickadee | Paridae | *Poecile rufescens* | 1 | 203 | 0.032201777 | 0.000484992 | 0.999515008 | **- |
| Pygmy Nuthatch | Sittidae | *Sitta pygmaea* | 1 | 242 | 0.038388325 | 8.00421E-05 | 0.999919958 | **- |
| White-crowned Sparrow | Emberizidae | *Zonotrichia leucophrys* | 1 | 249 | 0.039498731 | 5.77029E-05 | 0.999942297 | **- |
| American Robin | Turdidae | *Turdus migratorius* | 3 | 339 | 0.053775381 | 4.46742E-05 | 0.999955326 | **- |
| Red-winged Blackbird | Icteridae | *Agelaius phoeniceus* | 1 | 261 | 0.041402284 | 3.28454E-05 | 0.999967155 | **- |
| Brewer's Blackbird | Icteridae | *Euphagus cyanocephalus* | 25 | 1027 | 0.162912437 | 2.10482E-05 | 0.999978952 | **- |
| Song Sparrow | Emberizidae | *Melospiza melodia* | 5 | 435 | 0.069003807 | 1.82814E-05 | 0.999981719 | **- |
| Western Scrub-Jay | Corvidae | *Aphelocoma californica* | 0 | 224 | 0.035532995 | 1.50046E-05 | 0.999984995 | **- |
| Bushtit | Aegithalidae | *Psaltriparus minimus* | 1 | 399 | 0.063293147 | 4.16556E-08 | 0.999999958 | **- |
| Unknown |  |  | 1 |  |  |  |  |  |
|  |  | Total | 308 | 6280 | 1 |  |  |  |
