# Supplementary material for: Bird-Window Collisions at a West-Coast Urban Park Museum: Analyses of Bird Biology and Window Attributes from Golden Gate Park, San Francisco
Source: PLoS One. 2016 Jan 5;11(1):e0144600. doi: 10.1371/journal.pone.0144600 (PMC4701451; doi:10.1371/journal.pone.0144600)
Supplement: S2 Table — (DOCX) [file pone.0144600.s004.docx]

S2 Table: Table of bird species that struck windows non-fatally. Four species that only struck non-fatally are marked by *.

| Species | N |
| --- | --- |
| *Accipiter cooperii** | 1 |
| *Buteo jamaicensis** | 2 |
| *Calypte anna* | 18 |
| *Carpodacus mexicanus* | 2 |
| *Charadrius vociferous** | 1 |
| *Euphagus cyanocephalus* | 6 |
| *Hirundo rustica* | 1 |
| *Hummingbird sp.* | 2 |
| *Junco hyemalis* | 2 |
| *Melospiza lincolnii* | 1 |
| *Melospiza melodia* | 1 |
| *Selasphorus sasin* | 2 |
| *Setophaga coronata* | 3 |
| *Troglodytes troglodytes** | 1 |
| Unknown | 2 |
| *Zenaida macroura* | 1 |
| *TOTAL* | 46 |
